# Oil-in-water emulsion formulated with eucalyptus leaves extract inhibit influenza virus binding and replication in vitro

**DOI:** 10.3934/microbiol.2017.4.899

**Published:** 2017-11-07

**Authors:** Maryam Sadat Sadatrasul, Neda Fiezi, Nasir Ghasemian, Mohammad Shenagari, Saber Esmaeili, Ehsan Ollah Jazaeri, Asghar Abdoli, Abbas Jamali

**Affiliations:** 1 Pars Vaccine Technology Company, Pasteur Biotechnology Incubation Center, Karaj, Iran; 2 Department of Microbiology, Guilan University of Medical Sciences, Rasht, Iran; 3 Departments of Epidemiology and Biostatistics, Pasteur Institute of Iran, Tehran, Iran; 4 Department of Hepatitis and AIDS, Pasteur Institute of Iran, Tehran, Iran; 5 Departments of Influenza and Other Respiratory Viruses Research, Pasteur Institute of Iran, Tehran, Iran

**Keywords:** influenza virus, oil-in-water emulsion, eucalyptus leaves extract, CCID_50_

## Abstract

Throughout human history, the human-beings have been used different types of plants as antimicrobial agents in fight against infectious diseases. Influenza virus is one of the most common causes of respiratory infection and transmitted through direct contact with flu infected individuals and contaminated substances or droplets. In the current study, both oil-in-water and water-in-oil emulsions with hydroalcoholic extract of eucalyptus leaves (OLHE) were developed and their antiviral efficiency was evaluated. To doing so, Madin-Darbey Canine Kidney (MDCK) cells were treated with effective minimal cytotoxic concentration of the formulated emulsions. The treated cells were then infected with 50% cell culture infectious dose (100 CCID_50_) of the A/H1N1 virus (the swine flu). The viral titers were measured by hemagglutination (HA) and cell culture infectious dose 50% (CCID_50_) assays. Also, to check the virus binding inhibition via the formulated extract, the viruses were incubated with the formulated extracts. Our study showed that the oil-in-water emulsions formulated with 2% eucalyptus leaves extract inhibited virus replication completely when the cells were infected by 100 CCID_50_ and decreased HA titer up to four fold. Therefore, this formulation, may hold promising application to prevent influenza virus transmission through direct contact among children and passengers.

## 1. Introduction

More than 30,000 species of plants have been used in traditional medicines. But, this is only a small proportion of the plants in the world that have been assessed for potential pharmaceutical usage. It has been estimated that over 25% of medicament contain plant-derived ingredients yet. Approximately 80% of people in developing countries be certain about herbal products as their primary source of healthcare [1]. Many plants have been demonstrated that have robust antiviral activity and some of them routinely used to treat animals and people who infected viral infection [2]. Duggar and Armstrong reported the first plant inhibitors in 1925, they revealed that TMV replication was prevented by the different plants extracts [3]. Nature is still fertile and full of different types of plants having an antiviral effect and each area possess its own distinct variety of plants which are not obtainable in another place around the world which can exploit to viral infections [4].

Eucalyptus has been used in traditional medicine worldwide as anti-inflammatory mixtures for the symptoms of respiratory infections, such as sinus congestion, flu and common cold [5]. Inhalation of eucalyptus byproducts has been applied to cure pharyngitis, bronchitis, and sinusitis [6].

Ideal formulations for local administration of an active herbal extract are creams and lotions majorly oil-in-water (o/w) emulsions which spread easily on the skin without any detectable residue and adhere to the treated area without being sticky [7]. The aforementioned emulsions typically composed of an oil phase plus an aqueous phase. Both the oil and aqueous phase can be used as a carrier [8]. In the present study an oil-in-water emulsion composed of paraffin was used as a carrier.

The influenza viruses are major etiologic infectious agents of human respiratory tract inflicting considerable health and economic burden [9]. Influenza virus spread among humans via: (1) by direct physical contact with an infected individuals; (2) by contact with flu virus contaminated objects (called fomites, such as toys, doorknobs); and (3) by breathing of virus-laden aerosols [10],[11]. Influenza transmission can be reduced by using moisturizing emulsion containing the hydroalcoholic extract of eucalyptus. Herein, we evaluated the antiviral activity of hydroalcoholic extract of eucalyptus and tow type of oil-in-water (o/w) and water-in-oil (w/o) emulsions formulated with hydroalcoholic extract of eucalyptus on replication and binding of influenza virus.

## 2. Materials and Method

### 2.1. Plant material

The leaves of eucalyptus camaldulensis were collected and dried at room temperature, then powdered. Afterward, 1 g of finely powdered plant was mixed with 10 mL of 80% ethanol [12]. Then, different percentage of extract was tested on virus replication and cell culture. The 2% of extract was selected as the best effective dose and formulated in two different emulsions.

### 2.2. Preparation of emulsions

In this study, both oil-in-water (o/w) and water-in-oil emulsions (w/o) were prepared. The aqueous phase is composed of PBS and extract of eucalyptus and the oil phase formed using liquid paraffin. The SPAN 80 and TWEEN 80 were used as emulsifier as the following percentage for w/o formulation: liquid paraffin 50.9%, TWEEN 80 7.9%, SPAN 80 1.2%, extract of eucalyptus 2% and PBS 38%. o/w formulation constructed by liquid paraffin 28%, TWEEN 80 5%, SPAN 80 2%, extract of eucalyptus 2% and PBS 63% [13].

### 2.3. Cell culture and cytotoxicity assay

Madin-Darby canine kidney (MDCK) cell line (ATCC CCL-34) was provided by the National Cell Bank of Iran, Pasteur Institute of Iran and were maintained in complete Dulbecco's Modified Eagle's Medium (Gibco, Gaithersburg, MD) at 37 °C in a humidified 5% CO_2_ incubator.

In order to assay cell cytotoxicity, the cells were seeded at 5 × 10^4^ cells/well in 96-well microplates and the extract of eucalyptus and formulated emulsions were added with different concentrations to the cell culture and incubated for 48 h at 37 °C and 5% CO_2_. Then, 50% cytotoxic concentration (CC_50_) and effective minimal cytotoxic concentration (EMCC) was determined using the MTT assay.

### 2.4. MTT assay

Colorimetric MTT assay was carried out based on Levi Raphael, et al. protocol. Briefly, the supernatant of the confluent cells was replaced with 100 µl of 1× MTT. Following 2 hours incubation to release the color from the cells 100 µl of acidic isopropanol was added and optical density (OD) measured at 570 nm using ELISA reader (Stat Fax-200) [14].

### 2.5. Inhibitory effect on the virus titer

The same volume of influenza virus (100 CCID_50_) and EMCC of the formulated emulsions and extract were mixed and incubated at 37 °C for 1 h. To get rid of unabsorbed viruses after 1 h incubation, the cells were washed and DMEM medium containing TPCK was added (100 µl/well). The virus was tittered by HA assay and CCID_50_ using Karber formula [15].

### 2.6. Hemagglutination assay

To evaluate the replication of virus in cell culture, a volume of 50 µl culture supernatants were harvested and 2 fold serially dilated in 96-well U-shape microplates. Then, 0.5% Chicken red blood cells (cRBCs) were added in volume of 50:50 µl to each well. Following gentle agitation, the plates were incubated for 30 min at RT incubation one hour at room temperature. Precipitation of the RBCs confirmed the absence of the virus while complete hemagglutination verified the presence of the virus [16].

### 2.7. Measurement of virus infectivity titers

To evaluate the inhibitory effect of the aforementioned compounds, the replication of virus in cell culture measured by CCID_50_. The supernatant of infected cells was harvested and tenfold serial dilution of the supernatant (virus) inoculated into MDCK cell which were grown to 80% confluency. Three days post infection, the cells were scored for cytopathic effects and Karber method were applied to calculate the CCID_50_ values [17].

### 2.8. Data analysis

All experiments presented in this paper were performed in triplicates and repeated at least three times. The data were presented as means ± Standard Deviation (SD) and statistical analyses were carried out using the Graphpad Prism 6.01 software (GraphPad Software Inc, San Diego, CA, USA).

## 3. Results

### 3.1. Cell cytotoxic assay

**Figure 1.**
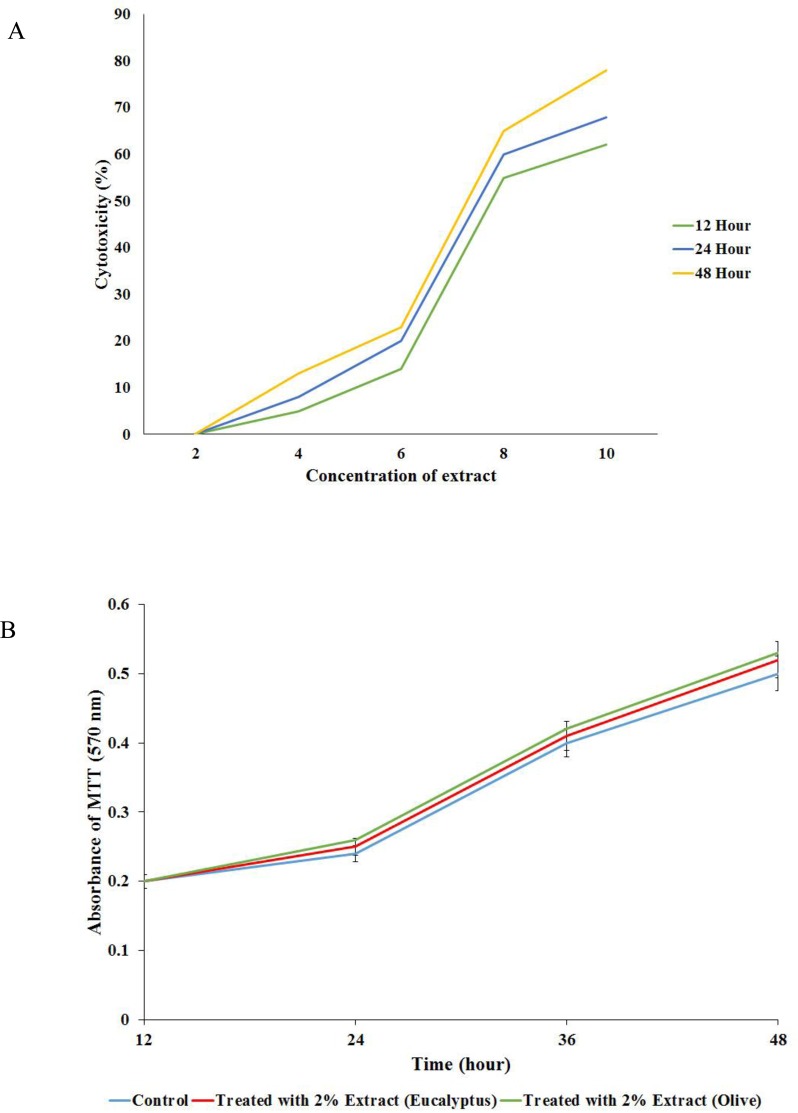
A: The 50% cytotoxic concentration (CC_50_) of eucalyptus leaves hydroalcoholic; B: effective minimal cytotoxic concentration (EMCC) of oil-in-water formulation of eucalyptus and olive extracts (as control).

The CC_50_ of eucalyptus leaves hydroalcoholic was 8% and effective minimal cytotoxic concentration (EMCC) using the MTT assay was 2% as shown in Figure 1A and Figure 1B.

### 3.2. Hemagglutination assay

**Figure 2.**
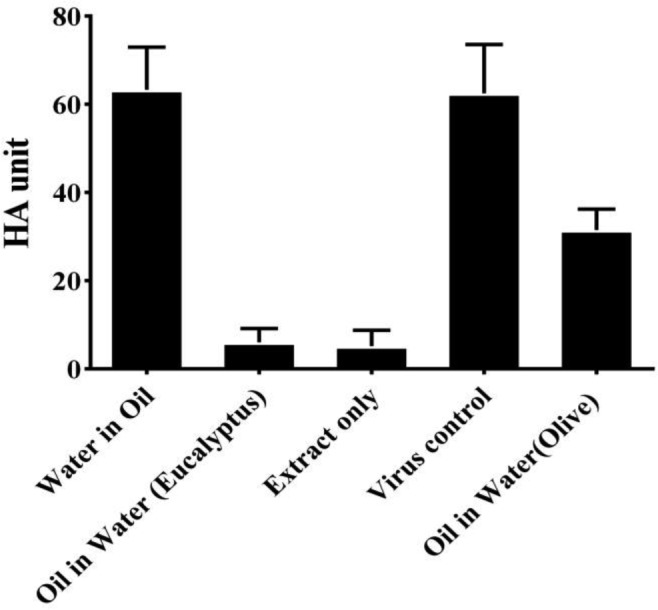
Effect of the eucalyptus leaves hydro alcoholic extract and formulated w/o and o/w emulsions on HA titer of influenza virus.

To determine whether the extract and formulated emulations interfere with the virus particle and virus binding in the ligand and receptor level, the HA assay was carried out. As depicted in Figure 2, the extract and o/w formulated emulsion significantly reduced H1N1 virus HA unit. But w/o formulated emulsion has not decreased the HA unit.

### 3.3. Virus infectivity titers

H1N1 titers were quantified in the H1N1-infected cell culture supernatants by a cell culture infectious dose 50% assay after 24 and 48 hours post infection. Table 1 shows the virus yield achieved in presence of eucalyptus leaves hydroalcoholic extract and the emulsions. After treatment of infected cell with extract and o/w there was no traces of infected virus, but w/o were not able to stop virus replication and virus titer was 10^4.5^ and 10^6.5^ 24 and 48 hours post infection, respectively.

**Table 1.** Antiviral activity of the eucalyptus leaves hydro alcoholic extract and formulated w/o and o/w emulsions of eucalyptus and olive on infective virus using CCID_50_.

Group	CCID_50_	
Time post-infection (hour)	24 h	48 h
Virus control	10^5^	10^6.5^
Oil-in-water (eucalyptus)	0	0
Water-in-oil	10^4.5^	10^6.5^
Extract only	0	0
Oil-in-water (Olive)	10^3^	10^3.5^

## 4. Conclusion

Developing new anti-influenza emulsion based herbal agent can play a key role in decreasing influenza virus direct or indirect contacts transmission [10]. Among the passengers who are seated near one another in public vehicle, child in school and farmer in field, flu virus can be communicated easily via direct or indirect contact. On the other hand, in contrast to spread through direct contact, host-to-host spread by way of respiratory droplets is severely sensitive to both temperature (30 °C) and relative humidity [18].

Although vaccination is the best choice to protect from influenza virus infections, this approach might be inefficient due to reassortaments and mutations that would change viral surface antigens. As a result, influenza virus be able to evade from immune response that have been already induced against vaccine antigens [19],[20]. On the other hand, only two anti-influenza drugs including inhibitors of the M2 ion channel (such as amantadine and rimantadine) and neuraminidase inhibitors (for example oseltamivir and zanamivir) have been approved; but treatment with these drugs may result in rapid emergence of resistant variants [21],[22],[23]. Therefore, finding an effective way to prevent or treat influenza disease is inevitable.

One of main evidence of an unspecific interference of plant extract with the virus particle rather than specific one is probably the reduced potentiality to arise resistant variants of influenza virus. So treatment with herbal formulated emulsions may be powerful approach in the case of the variable influenza virus [24]. In the current study, on a molecular basis, the formulated extract seems to interfere with the virus particle and virus attachment, since viral agglutination of RBC was affected significantly in the presence of antiviral concentrations of formulated extract. Sawai and colleagues indicated that high molecular weight polyphenols present in the fruits of *C. sinensis* could neutralize influenza virus by inhibiting hemagglutination activity and suppressing NS2 protein synthesis [25]. Also, Roschek and coworkers isolated two anti-influenza flavonoids from an optimized elderberry (Sambucus nigra) fruit extract and showed that the isolated flavonoids bind to H1N1 virions and block viruses infecting host cells [26]. Hamauzu and co-workers suggested that the phenolic extract of *C. sinensis*, inhibits the hemagglutination activity of the influenza virus and halts the first step of the infection by disabling the virus's ability to adhere to host cells [27].

Eucalyptus leaf extract has been used as “remedy” for the symptoms of cold and flu in some folk medicines [28]. Compounds found in eucalyptus leaf have direct microbicidal activity against bacteria, viruses, mycobacteria and fungi. The biological activities of eucalyptus leaf are mainly derived from mixture of compounds such as flavonoids and biophenols [29]. Therefore ,the present of antimicrobial compound in the extract of eucalyptus leaf add another favor to acting against bacterial co-infections, alternative life threatening complication in severe influenza virus disease [30].

Our results indicate that, in addition to prevent virus attachment, the formulated extract be able to block virus replication in MDCK cells when cell infected with 100 CCID_50_. Polyphenols bear protein-binding capacity, probably also physically interact with pathogens and/or cellular surfaces in an unspecific manner. Ehrhardt et al. [24] showed that the LADANIA067 content in polyphenols seems to interfere with the virus particles during virus internalization, resulting in reduced viral uptake into the host cells.

Emulsions are well-known carrier systems in the pharmaceutical industry for the properties that allow them to convey their content to the treated area [31]. In this study, in contrast to o/w the w/o was not efficient in blocking virus replication. Because in o/w emulsion, the continuous phase is composed of the water and the dispersed phase is the oil while in a w/o emulsion the oil form the continuous phase [32]. The herbal extract is soluble in water, when the w/o was used the oil is continuing phase and mask the extract; therefore, prohibit the contact of extract and virus. That is the reason why w/o formulation was not able to block virus replication

Plant extracts have been used for centuries in traditional medicine without reports of adverse effects or allergic reactions, due to the plant extract has no specific activity, and thus, they may not lead to emerge of resistance viral strains. In addition, the co-infection bacterial agent would be reduced by application of herbal compound in sever influenza disease. Taken together, with respect to the simplicity contagious of influenza infection through direct contact with infected individuals and contaminated objects, formulation of eucalyptus leaf extract with oil-in-water carrier as lotion, cream or syrup may be an efficient strategy to prevent influenza virus spreading through direct and indirect contact among children and passengers (in public places) and even a flu epidemic consequently.
